# Neonatal spontaneous pneumomediastinum and the Spinnaker-Sail sign

**DOI:** 10.1590/S1679-45082015AI3133

**Published:** 2015

**Authors:** Ricardo Monteiro, Lígia Paulos, João do Agro, Lina Winckler

**Affiliations:** 1 Centro Hospitalar Leiria Pombal, Leiria, Portugal.

Pneumomediastinum in full-term newborn is associated with meconium aspiration syndrome, hyaline membrane disease, mechanical ventilation or traumas related with labor. The spontaneous neonatal pneunomediastinum is rare. We present imaging exams crucial for the diagnosis of a clinical case of pneumomediastinum in a newborn.^(1,2)^

**Figure 1:**
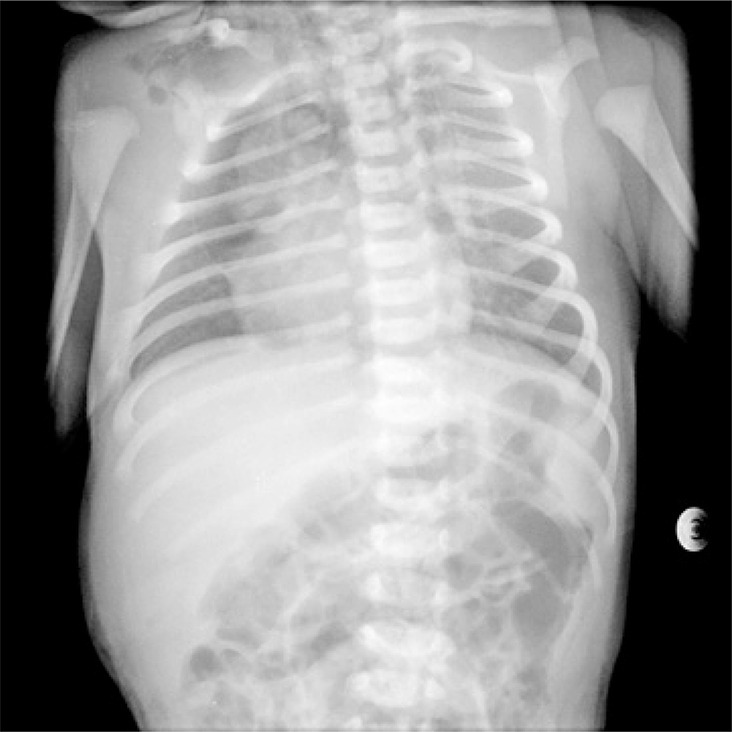
Chest radiography showing Spinnaker-Sail sign (wedge-shaped opacity extending to superior mediastinum, inferiorly delimited by a hyper-transparent area)

**Figure 2:**
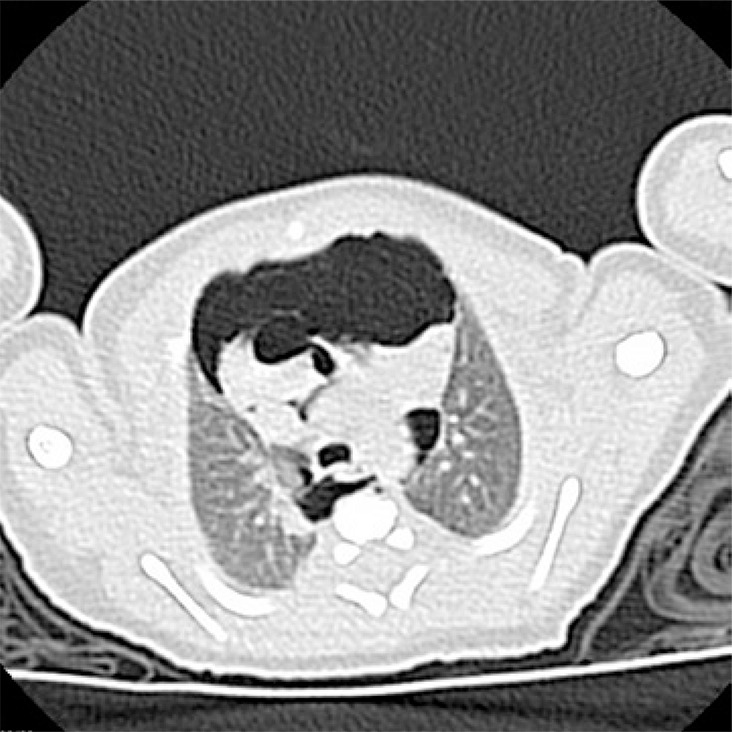
Computed tomography showing an extensive pneumomediastinum with mild septation, unchanged lung parenchyma (cross-sectional cut)

**Figure 3:**
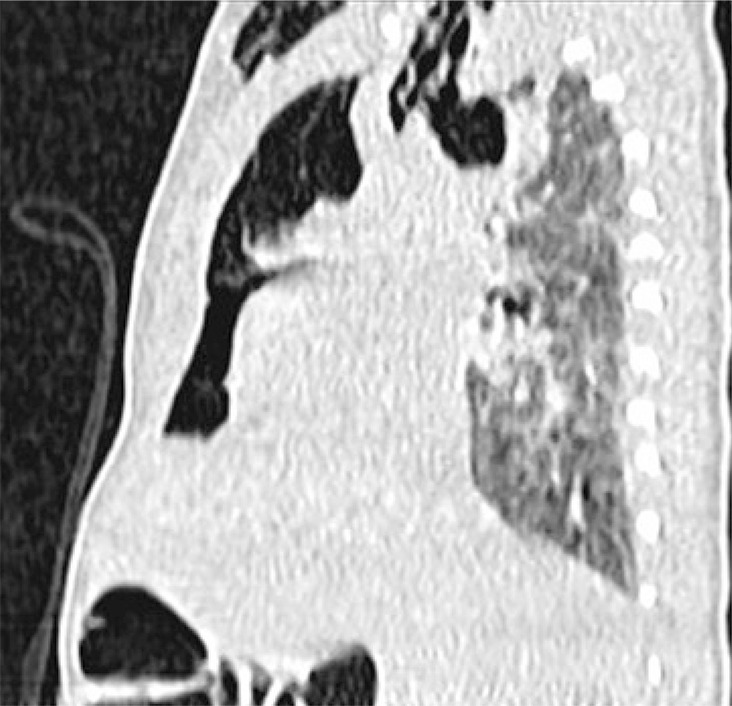
Computed tomography showing subcutaneous enphysema and extensive pneumomediastinum with mild septation, unchanged lung parenchyma (sagittal cut)

This is a radiology exam of a newborn whose mother pregnancy was monitored and occurred without intercurrences. The infant born at 38 weeks of gestation by vacuum assisted delivery, immediate crying and Apgar score of 9/10. The diagnosis of subcutaneous emphysema in anterosuperior portion of the thorax occurred in the infant first day of life. Chest radiography showed subcutaneous cervical emphysema and Spinnaker-Sail sign (wedge-shaped opacity extending to superior mediastinum, inferiorly delimited by a hyper-transparent area) (Figure 1). Newborn’s computed tomography showed subcutaneous emphysema and extensive pneumomediastinum, mild septation and unchanged lung parenchyma (Figures 2 and 3). In addition, the baby was always stable and had good clinical progress.

This clinical report highlights the important role of chest radiography in the diagnosis of most of pneumomediastinum cases. The Spinnaker-Sail sign (an image resembling the headsail of a boat, translating the lateral displacement of thymus in relation to pericardium caused by the air) is a pathognomonic sign of pneumomediastinum.^(1,3,4)^

It is important to emphasize that the image of “headsail of a boat” in radiography cannot be totally well-defined and other congenital pathologies can be assumed. Anatomical characteristics of newborns’ thymus must be considered when pneumomediastinum is suspected, particularly because such characteristics can pose difficulties to interpret the exam. Hence, chest computed tomography is crucial for the adequate diagnosis.^(1-4)^
