# Supplementary material for: Neonatal spontaneous pneumomediastinum and the Spinnaker-Sail sign
Source: Einstein (Sao Paulo). 2015 Oct-Dec;13(4):642–3. doi: 10.1590/S1679-45082015AI3133 (PMC4878646; doi:10.1590/S1679-45082015AI3133)
Supplement: Translation

# Translation: Pneumomediastino espontâneo neonatal e o sinal de Spinnaker

**Authors:** Ricardo Monteiro, Lígia Paulos, João do Agro, Lina Winckler

**Affiliations:** 1 Centro Hospitalar Leiria Pombal, Leiria, Portugal.

O pneumomediastino num recém-nascido de termo é associado a aspiração meconial, pneumonia, doença das membranas hialinas, ventilação mecânica ou a traumatismos relacionados com o parto. O pneumomediastino espontâneo em recém-nascido é raro. A seguir, apresentamos os exames imagiológicos fundamentais para o diagnóstico num caso clínico de pneumomediastino em recém-nascido.^(1,2)^

O exame radiológico apresentado pertence a um recém-nascido cuja gravidez foi vigiada e decorreu sem intercorrências. Nasceu por ventosa com 38 semanas, com choro imediato e índice de Apgar 9/10. Foi diagnosticado, no primeiro dia de vida, com enfisema subcutâneo na face anterossuperior do tórax. Efetuou uma radiografia de tórax que mostrou enfisema subcutâneo cervical e sinal de “vela de Spinnaker” (cunha hipotransparente estendendo-se até ao mediastino superior delimitada inferiormente por zona hipertransparente) (Figura 1). Fez tomografia computorizada, que mostrou enfisema subcutâneo e extenso pneumomediastino, com discreta septação e sem alterações do parênquima pulmonar (Figuras 2 e 3). O recém-nascido esteve sempre estável e apresentando boa evolução clínica. Controle radiológico no sétimo dia de vida sem alterações.

**Figura 1:** Radiografia de tórax mostrando o sinal de “vela de Spinnaker” (cunha hipotransparente que se estende até ao mediastino superior, delimitada inferiormente por zona hipertransparente)

**Figura 2:** Tomografia computorizada que mostra extenso pneumomediastino com discreta septação, sem alterações do parênquima pulmonar (corte transversal)

**Figura 3:** Tomografia computorizada que mostra enfisema subcutâneo e extenso pneumomediastino com discreta septação, sem alterações do parênquima pulmonar (corte sagital)

Discute-se, neste caso clínico, o papel preponderante da radiografia de tórax no diagnóstico da maioria dos casos de pneumomediastino. O sinal da “vela de Spinnaker” (imagem semelhante à vela de um barco, traduzindo a dissecção lateral do timo em relação ao pericárdio pelo ar) é patognomônico de pneumomediastino.^(1,3,4)^

Realça-se que, conforme é visível na radiografia apresentada, a imagem da “vela do barco” pode não ser totalmente bem definida e suscitar dúvidas relativamente à possibilidade de outras patologias congênitas. As características anatômicas do timo neonatal devem ser tidas em linha de conta, face à suspeita de clínica de pneumomediastino, uma vez que este pode dificultar a interpretação do exame. Nessas circunstâncias, a realização da tomografia computorizada torácica é fundamental para estabelecer o diagnóstico.^(1-4)^
